# Decreased psychiatric symptomatology after the onset of COVID-19 in a longitudinal college mental health study

**DOI:** 10.1038/s44184-022-00017-4

**Published:** 2022-10-21

**Authors:** Asli Ercan Dogan, Dilek Kebapci, Oguz Ertan, Zeynepgul Kalay, Nurdan Kozan Caki, Vedat Sar, Hale Yapici Eser

**Affiliations:** 1 Department of Psychiatry, Koç University, School of Medicine, İstanbul, Turkey; 2 Department of Psychiatry, Koç University Hospital, İstanbul, Turkey; 3 Koç University, School of Medicine, İstanbul, Turkey; 4 Koç University Psychological Counselling and Psychotherapy Center (KUPTEM), İstanbul, Turkey; 5 Research Center for Translational Medicine, Koç University, İstanbul, Turkey

**Keywords:** Medical research, Risk factors

## Abstract

The COVID-19 pandemic brings significant challenges for college students. This study aims to investigate changes in psychiatric symptomatology among them compared to the pre-pandemic period alongside their determinants. Data are collected before and 3 months after the onset of the pandemic from 168 students who applied to a college mental health center. Psychiatric symptomatology is assessed by the Patient Health Questionnaire-9 (PHQ-9), Generalized Anxiety Disorder-7 (GAD-7), and Adult Attention Deficit Hyperactivity Disorder Self-Report Scale (ASRS). Possible vulnerability factors are screened by a survey on COVID-19-related health and social isolation status, Fear of COVID-19 Scale, Social Media Use Disorder Scale (SMDS), Distress Thermometer, Scoff Eating Questionnaire, and International Physical Activity Questionnaire Short-Form (IPAQ). Results show decreased PHQ-9, GAD-7, and ASRS scores at follow-up. Even though the screen time increases, SMDS scores significantly decline. SMDS have a direct effect on PHQ-9 and ASRS levels, in addition to an indirect effect through the Distress Thermometer. Higher SMDS scores predict higher anxious and depressive symptomatology in repeated assessments. Fear of COVID-19 scores have a direct effect on GAD-7 scores only. This study suggests that the stress level and psychiatric symptomatology of the students decreased significantly in the early phases of the pandemic. The level of social media use disorder should be taken into account while following college students with mental health symptoms.

## Introduction

The world is going through a global pandemic of coronavirus disease 2019 (COVID-19), which is causing significant consequences for healthcare systems and public health. Studies have reported that spatial distancing, self-isolation, quarantine, social and economic discord, and misinformation (particularly on social media) are among the major contributing factors to unusual sadness, fear, frustration, feelings of helplessness, loneliness, and nervousness^1,2^. A recent systematic review concluded that the COVID-19 pandemic is associated with highly significant levels of psychological distress which meets the threshold for depression, anxiety disorders, and posttraumatic stress disorder in many cases^3^. College students are an emerging focus among vulnerable groups for the psychiatric impact of the pandemic. They are in an age of emerging adulthood (18–25 years of age) which is a second transition period of life after adolescence when well-being can easily become unstable especially during extraordinary circumstances^4^. Worldwide data collected before the pandemic suggest that approximately 20% of college students experience at least one diagnosable mental disorder^5^. The closure of university campuses with the COVID-19 pandemic brought significant challenges to college students’ daily routines. Prolonged social isolation, a quick change in educational environment^6^, the fear of getting infected and not being able to meet their families for an unknown duration^7^, anxiety about the potential impact of the pandemic on their studies and future job market opportunities^8^ are leading risk factors according to early studies. In addition, lifestyle and health behavior changes such as eating and drinking habits, sleep quality, physical activity, smartphone, and social media use may also have an impact on students’ mental health^9^. While social media may cause the rapid spread of false information and rumors that create panic and confusion in the public, it can also play a positive role in information exchange in times of crisis such as COVID-19^10^. College students who had to stay at home during the pandemic used social media to learn and communicate which increased the duration and frequency of social media use. Since the symptoms of problematic social media use are spending too much time and preoccupation, excessive use of social media can easily turn into problematic use^11^. Although some studies reported a positive influence of social media use on mental health, problematic social media use has been linked to poor psychological well-being and symptoms of depression and anxiety^12,13^. A study from China during the COVID-19 pandemic showed that problematic social media use among university students predicted their levels of anxiety^14^.

A large number of cross-sectional studies reported varying degrees of increased acute stress, anxiety, and depressive symptoms in college students. For example, a nationwide survey of college students in China has found increased rates of acute stress, depressive, and anxiety symptoms, which were 34.9, 21.1, and 11.0%, respectively^15^. Another survey from the USA reported that nearly half of the students showed a moderate-to-severe level of depression and nearly 40% of students showed a moderate-to-severe level of anxiety^16^.

However, cross-sectional studies neither explain how students’ mental health has changed compared to pre-pandemic levels nor identify the predictors of this change. A few prospective studies have been published so far reporting mixed results on the change in psychiatric symptomatology in college student populations^8,17–19^. There is a need for studies investigating the effects of the COVID-19 pandemic on the mental health of university students and its related factors such as lifestyle changes and social media use. Thus, we aimed to investigate (1) the changes in psychiatric symptomatology of the students compared to the pre-pandemic period, (2) the determinants of this change in relation to COVID-19 exposure, social isolation status, lifestyle changes, stress levels, and social media use use disorder, (3) the correlations of follow-up psychiatric measurements with each other, and (4) the potential mediating effect of stress on depression and anxiety levels.

As a result of the analyses, compared to the pre-pandemic period, students are found to have decreased symptoms of depression, anxiety, and attention deficit. The decrease in stress levels and symptoms of social media use disorder are the predictors of this change. The level of stress is mediating the relationship between (i) social media use disorder and depression, (ii) social media use disorder and attention deficit, and (iii) fear of COVID-19 and anxiety.

## Methods

### Study setting

Turkey, with >15 million cases, is among the top 10 countries in the world where COVID-19 has spread the most, as of June 2022. The first COVID-19 positive case in Turkey was announced on March 11, 2020, and by 1 April it was confirmed that COVID-19 had spread all over the country. On March 16, 2020, face-to-face education was suspended in all universities. From the beginning of April 2020, some additional regulations have been made by the Turkish government such as the restriction of intercity travel, the closure of shopping centers, cinemas, restaurants, and sports centers, and a lockdown for people under the age of 20 years. By the end of March 2020, all Koc University campuses were evacuated and the transition to online education was completed.

This study was conducted in the Koc University Psychological Counseling and Psychotherapy Center, which is a comprehensive mental health center that provides various clinical services including individual psychotherapy, psychiatric consultation, and psychiatric treatment. The center accepts applications by email all year round.

### Study cohort

This study was approved by the Koc University Institutional Review Board and all procedures were in accordance with the Declaration of Helsinki. All participants provided an online informed consent before participating in the study.

Our target population was college students who already had psychiatric problems before the pandemic. The accessible population was the college students who applied to our college mental health center during the pre-pandemic period. We invited the whole accessible population to the study, 330 students who applied to the Koc University Psychological Counseling and Psychotherapy Center between September 2019 and March 2020 and completed the pre-evaluation scales. Therefore, the sampling method was convenient sampling. Students were contacted via their e-mail addresses which they gave consent to be contacted at their first application. Students were informed about the aims and steps of the study and invited to take the online survey for reassessment. A discount coupon was offered as a compensation upon participating in the study. If students did not respond to the first e-mail, a reminder email was sent within a week. Follow-up data were collected between June 11 and June 22, 2020.

Overall, 168 students participated in the study and completed the surveys. Due to the longevity of the scales, students were given the right to end the survey before answering all scales. One hundred and thirty-three students completed the whole scales and questionnaires.

**Fig. 1:**
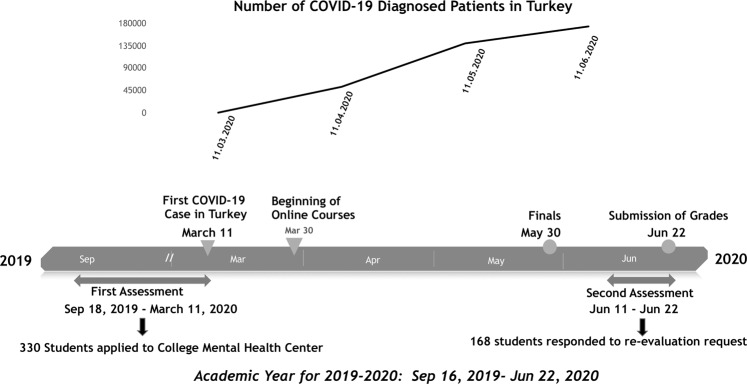
Study timeline in relation to national COVID-19 cases and academic calendar. The timing of the first and second assessment periods are given together with the university academic calendar and the course of the number of COVID-19 cases in Turkey.

The characteristics of the study sample (*n* = 168) are given in the “Results” section. The timeline of pre-pandemic assessment, the onset of COVID-19 in Turkey, the academic calendar of the university, and data recruitment dates are presented in Fig. 1.

### Evaluation scales

Both pre-pandemic and follow-up surveys were administered online. Students are sent an online Qualtrics survey link, and they complete the survey as a self-report. Each online survey included attention questions to increase the reliability of the data collected. The online survey included four parts questioning both pandemic-related variables and psychiatric symptomatology.

#### Survey on demographic and health behavior data

This survey questioned the demographic variables, living conditions, sleep habits, and whether they received psychotherapy or psychiatric treatment between pre-pandemic and pandemic measurements. Psychotherapy is defined as having a minimum of four psychotherapy sessions and psychiatric treatment is defined as having a regular pharmacotherapy regimen for at least 3 months.

#### Measurements on COVID-19 pandemic process

##### Health issues associated with the COVID-19 pandemic survey

This survey questioned whether students or their relatives developed physical symptoms related to COVID-19 or were diagnosed with COVID-19, if so, how they were treated (such as outpatient, inpatient service, intensive care), whether any of their relatives passed away due to COVID-19 in the period since the pandemic was announced in Turkey until the day that they filled out the survey.

##### Survey on social isolation during the COVID-19 pandemic

This survey questioned where and with whom students live, the degree of social isolation (how many times they leave home, how many people they have average contact with, how often they use social media for socializing) in the period since the pandemic was announced in Turkey until the day that they filled out the survey.

##### The Fear of COVID-19 Scale

It is a 7-item five-point Likert-type scale questioning anxious thoughts and physical symptoms related to anxiety; higher scores indicate a greater level of anxiety. The overall score of fear (ranging from 7 to 35) was obtained by adding up each item score^20^. It is developed by Ahorsu et al. to measure the fear of COVID-19. The original English version of the scale is used in this study.

#### Measurements of psychiatric symptomatology and functionality

##### Patient Health Questionnaire-9

It was developed by Kroenke, Spitzer, and Williams for diagnosing depression and other mental disorders based on criteria, which are frequently encountered in intensive care^21^. It has 9 questions, and is answered by scoring between 0 and 3. The Turkish validity and reliability study was conducted by Sari et al.

##### Generalized Anxiety Disorder-7

It was developed by Spitzer, Kroenke, Williams, and Lowe as a brief clinical scale in order to evaluate generalized anxiety disorder^22^. The scale with 7 questions is answered by scoring between 0 and 3. The Turkish validity and reliability study was conducted by Konkan, Senormanci, Guclu, Aydin, and Sungur.

##### Adult Attention Deficit Hyperactivity Disorder (ADHD) Self-Report Scale (ASRS-v1.1)

It was developed in collaboration with the World Health Organization and Adult ADHD Study Group in 2005^23^. It has been developed as a tool that can be used to evaluate adult patients in terms of ADHD. It consists of 18 questions, is answered in a 5-Likert type range in the range of “Never,” “Rarely,” “Sometimes,” “Often,” and “Very often.” The Turkish validity and reliability study was conducted by Dogan, Oncu, Varol-Saracoglu, and Kucukgoncu.

##### World Health Organization Disability Assessment Chart (2010)

It aims to evaluate the health status and disability independent from medical diagnosis. It is a 12-item scale and answered in 5-point Likert type. The Turkish validity and reliability study was conducted by Kunt and Dereboy.

#### Measurements related to risk factors

##### Distress Thermometer

It is developed by Roth et al. with 1 question and answered as a 10-point scale between “0 = no distress” and “10 = distress at the highest limit”^24^. The Turkish validity and reliability study was conducted by Ozalp, Cankurtaran, Soygur, Geyik, and Jacobsen.

##### Social Media Use Disorder Scale-9

It was developed by Van den Eijnden, Lemmens, and Valkenburg, which aims to measure adolescents’ level of social media addiction^25^. It is a scale with 9 questions and answered as an 8-point scale between “0 = Never” and “7 = More than 40 times a day.” The Turkish validity and reliability study was conducted by Saricam and Adam Karduz.

##### The SCOFF questionnare

It was developed by Morgan, Reid, and Lacey to screen eating disorders. It is a scale with 5 questions and answered as “Yes” or “No”^26^. The Turkish validity and reliability study was conducted by Aydemir, Koksal, Yalin Sapmaz, and Yuceyar.

##### International Physical Activity Survey

This questionnaire was developed between 1997 and 1998 by a group of physical activity assessors known as the International Consensus Group in the study by Craig et al. in order to observe international physical activity and inactivity^27^. The short form contains 7 short open-ended questions. In addition, Questions 2, 4, and 6 were designed as conditional questions for questions 1, 3, and 5, respectively. The Turkish validity and reliability study of the questionnaire was conducted by Memis F.

Except for the Fear of COVID-19 Scale, the Turkish versions of all scales^28–34^ were used in the study.

### Statistical analysis

Pre-pandemic and follow-up measurements for continuous variables were compared with the paired samples *t* test, and the non-parametric alternative Wilcoxon signed-rank test was used for dependent variables that were not normally distributed. Due to multiple comparisons of various variables, we used a corrected *p* value. Since the number of tests was 8 in our study, a *p* value lower than 0.006 was accepted as significant for this analysis. Cohen’s *d* (calculated with *t*/√*N*) was used for effect size calculations. Independent samples *t* test and the non-parametric alternative Mann–Whitney *U*-test were used for the comparisons of continuous variables between respondents and non-respondents. Distributions of categorical variables in pre-pandemic and follow-up assessments were compared by the McNemar test.

To determine which factors affected the decrease in psychiatric symptomatology, we specified two intercept-only models to define the predictor variables for each outcome variable (PHQ-9, GAD-7, and ASRS), in a linear mixed effect model where the outcome variable was regressed on a random effect variable for participants, and all other variables were defined as fixed factors, using Jamovi Version 1.1.9.0. All other analyses were conducted using IBM SPSS Statistics for Windows, Version 26.0. Armonk, NY: IBM Corp. Program.

To test the interaction between Fear of COVID-19 Scale, Distress Thermometer, SMDS, and psychiatric outcome (PHQ-9, GAD-7, and ASRS) scores, we conducted mediation analysis with model 4 under PROCESS macro version v3.5, developed by Hayes for SPSS. In the mediation models, fear of COVID-19 scores and SMDS scores were defined as *X*, and psychiatric outcome (PHQ-9, GAD-7, and ASRS) scores were defined as *Y*, whereas Distress Thermometer scores were defined as the mediators. For this analysis, only participants’ scores that were complete for all scales (*n* = 151) in the second assessment were included in the model, as this analysis required cross-sectional data. The mediation analysis is performed by the bootstrapping method with 5000 samples and bias-corrected 95% confidence intervals (CIs) to estimate the significance of the indirect and direct effects.

### Reporting summary

Further information on research design is available in the Nature Research Reporting Summary linked to this article.

## Results

### Demographic variables, health behavior, and COVID-19-related status of the participants

A total number of 330 students were asked to participate and 168 of them participated in the study. Therefore, the response rate was 50.9%. A Mann–Whitney *U*-test revealed no differences in baseline stress levels, PHQ-9, GAD-7, ASRS scores, or psychosocial functioning between responders and non-responders (*p* > 0.05).

Twenty-one percent of the applicants (*n* = 168) were referred by university counseling service or academic staff without a formal diagnosis, and the rest stated that they heard about our center through the university website, orientation training, and from peers. The most common reasons for application in the pre-pandemic period were: 60% depressive and anxious symptoms, 30% difficulty in fulfilling academic responsibilities, 32% difficulties in social relationships, 24% psychosomatic symptoms, 18% romantic relationship problems, 16% family problems, and 16% obsessive-compulsive symptoms.

A total of 120 (71.4%) of the participants were female. The mean age of the participants was 21.7 ± 2.52. The number of days of alcohol use decreased significantly (*p* < 0.001*). Screen time increased significantly (*p* < 0.001*); for example, the frequency of screen time of 7 h or more per day was 13.2% before the pandemic, while this rate was found 38.6% during the pandemic period. Seventy-seven of the participants (45.8%) received psychotherapy or psychiatric management in between the two assessments. The average number of days between pre-pandemic and follow-up assessments was 190.66 (min 94–max 275). The rate of living with parents increased significantly from 4.8 to 38% (*p* < 0.001*). The details of these data can be found in Supplementary Table 1.

The mean score of the fear of COVID-19 scale was 17.23 ± 5.43. While 18.9% of participants experienced some possible COVID-19-related symptoms, 81.1% of them stated that they did not experience any symptoms related to COVID-19. A total of 44.9% of participants isolated themselves that they did not expose themselves to anyone other than housemates. A total of 83.7% of the students were accompanied by their families during the lockdown, and the rest were either alone (3.7%) or with their friends (12.6%) (Supplementary Table 2) (*McNemar test).

### Change in psychiatric symptomatology during the pandemic

The percentage of students who had at least moderate depressive symptoms (phq-9 score ≥10) was 74.3% in the pre-pandemic period while this rate was found 52.3% in the follow-up. In the pre-pandemic period, 59.6% of students showed moderate to severe anxiety symptoms (gad-7 score ≥10) while this rate was found 36.4% in the follow-up. The frequency tables related to pre-pandemic and follow-up PHQ-9 and GAD-7 scores can be found in Supplementary Tables 3–6. The comparison of Distress Thermometer, PHQ-9, GAD-7, ASRS, SMDS, IPAQ, SCOFF, and WHODAS scores between the pre-pandemic and follow-up period is presented in Table 1. GAD-7, PHQ-9, and ASRS scores did not differ between those who received psychotherapy or psychiatric treatment and those who did not (*p* > 0.05**) (**Mann–Whitney *U*-test).

**Table 1:** Comparison of pre-pandemic and follow-up psychiatric assessments.

Scale	Pre-pandemic assessment	Follow-up assessment	95% CI of the difference	*t*	*p* value	Cohen’s *d*
PHQ-9 (*n* = 149)	13.47 ± 5.40	11.17 ± 5.36	1.36–3.22	4.89	<0.0001	0.40
GAD-7 (*n* = 149)	10.95 ± 5.39	8.31 ± 4.97	1.69/3.59	5.51	<0.0001	0.45
Distress Thermometer (*n* = 154)	64.29 ± 22.47	52.19 ± 25.24	7.45/16.75	5.14	<0.001	0.41
SMDS (*n* = 138)	23.21 ± 8.09	18.12 ± 5.72	4.01/6.15	−2.40	<0.0001	0.20
ASRS (*n* = 146)	34.16 ± 11.22	31.68 ± 11.75	0.86/4.09	3.03	0.003	0.28
WHODAS (*n* = 145)	15.76 ± 7.92	14.06 ± 8.65	0.30/3.10	2.39	0.018	0.20
SCOFF (*n* = 150)	1.04 ± 1.16	1.24 ± 1.26	−0.36/−0.03	−2.34	0.02	0.19
IPAQ (*n* = 168)	1769.67 ± 1757.8	1679.73 ± 1521.20	−236.7/416.6	0.54	0.59	0.04

Note: A *p* value < 0.006 was considered significant due to multiple comparisons.

*p* values were calculated by using Wilcoxon signed-rank test.

### Determinants of improvement in psychiatric symptomatology

To determine which factors affected the decrease in psychiatric symptomatology, we specified two intercept-only models to define the predictor variables for each outcome variable (PHQ-9, GAD-7, and ASRS), in a mixed effect model where the outcome variable was regressed on a random effect variable for participants, and all other variables were defined as fixed factors, using the JAMOVI package. In the first model, the health behaviors that significantly changed during the pandemic were included: living with parents (as a factor), screen time, and the number of days of alcohol use in the last month (as covariates). In the second model, Distress Thermometer and SMDS scores were added to the model as covariates. The results of the linear mixed effects model analysis are presented in Table 2. Both higher Distress Thermometer and SMDS scores predicted higher PHQ-9, GAD-7, and ASRS scores in the mixed effect regression model, where pre-pandemic and follow-up scores were used (Table 2). Living with parents and alcohol use were significant predictors of both PHQ-9 and GAD-7; however, their effects became non-significant in the second model where Distress Thermometer and SMDS scores were included as covariates.

**Table 2:** Mixed effect regression of changed variables on PHQ-9, GAD-7, and ASRS scores.

	PHQ-9						GAD-7						ASRS					
	Block 1			Block 2			Block 1			Block 2			Block 1			Block 2		
	*B*	95 % CI	*p*	*B*	95 % CI	*p*	*B*	95 % CI	*p*	*B*	95 % CI	*p*	*B*	95 % CI	*p*	*B*	95 % CI	*p*
Intercept	11.86	11 to 12.7	<0.001	12.25	11.53 to 12.97	<0.001	9.3	8.45 to 10.14	<0.001	9.67	9 to 10.34	<0.001	33.1	31.2 to 35	<0.001	33.78	32.1 to 35.5	<0.001
Living with parents	−1	−1.73 to −0.22	0.01	−0.26	−0.92 to 0.38	0.42	−0.71	−1.5 to 0.06	0.072	−0.03	−0.67 to 0.61	0.92	−0.13	−1.5 to 1.3	0.86	1.06	−0.27 to 2.4	0.12
Screen time	−0.15	−0.97 to 0.67	0.71	−0.21	−0.9 to 0.47	0.54	−0.47	−1.3 to 0.35	0.27	−0.6	−1.27 to 0.08	0.09	0.51	−1.04 to 2.05	0.52	0.56	−0.86 to 1.99	0.43
Alcohol use	0.52	0.10 to 0.94	0.015	0.25	−0.1 to 0.60	0.16	0.44	0.02 to 0.85	0.04	0.14	−0.2 to 0.48	0.42	0.93	0.12 to 1.74	0.025	0.56	=−.18 to 1.31	0.14
Stress thermometer				0.1	0.078 to 0.12	<0.001				0.11	0.09 to 0.13	<0.001				0.11	0.06 to 0.15	<0.001
SMDS				0.15	0.08 to 0.23	<0.001				0.11	0.04 to 0.19	0.003				0.35	0.18 to 0.51	<0.001
Random parts																		
AIC	1623.62			1533.3			1621			1524			1948			1908		
R2 marginal	0.053			0.33			0.04			0.36			0.0175			0.157		
R2 conditional	0.45			0.58			0.36			0.52			0.67			0.66		
Number of observations	266			266			266			266			263			263		
Groups	133			133			133			133			133			133		
ICC	0.42			0.37			0.33			0.25			0.62			0.59		

### Relationship of fear of COVID-19 with current psychiatric status

Table 3 shows the correlations of follow-up psychiatric assessments including the correlations between Fear of COVID-19 and other psychiatric symptoms. A multivariate linear regression analysis was carried out to investigate the predictors of current Distress Thermometer scores by putting the fear of COVID-19, PHQ-9, and GAD-7 scores in the model. This analysis revealed that the model explained 40.6% of the variance and that the model was a significant predictor of Distress Thermometer scores [(*F* (3,150) = 33.56, *p* < 0.001]. While PHQ-9 (*B* = 1.03, *p* = 0.009) and GAD-7 (*B* = 2.25, *p* < 0.001) scores significantly predicted Distress Thermometer scores, Fear of COVID-19 scores (*B* = 0.39, *p* = 0.215) did not.

**Table 3:** Correlations of follow-up (pandemic) psychiatric assessments.

	Fear of COVID-19	PHQ-9	GAD-7	Stress Thermometer	SMDS	ASRS	WHODAS
Fear of COVID-19	1	0.215**	0.299**	0.277**	0.238**	0.213**	0.355**
PHQ-9	0.215**	1	0.651**	0.525**	0.337**	0.570**	0.525**
GAD-7	0.299**	0.651**	1	0.610**	0.202*	0.434**	0.366**
Stress Thermometer	0.277**	0.525**	0.610**	1	0.202*	0.285**	0.359**
SMDS	0.238**	0.337**	0.202*	0.202*	1	0.377**	0.386**
ASRS	0.213**	0.570**	0.434**	0.285**	0.377**	1	0.440**
WHODAS	0.355**	0.525**	0.366**	0.359**	0.386**	0.440**	1

Spearman rank correlation test was used to calculate correlation coefficients.

**Correlation is significant at the 0.01 level (2-tailed).

*Correlation is significant at the 0.05 level (2-tailed).

### Mediation of PHQ-9, GAD-7, and ASRS scores by Distress Thermometer, Fear of COVID-19, and SMDS scores

We have predicted that the Distress Thermometer scores would have a mediating effect on GAD-7 and PHQ-9 scores. SMDS and Fear of COVID-19 scores seemed to have both direct and indirect effects on GAD-7 and PHQ-9 scores via Distress Thermometer scores. When SMDS (*X*), PHQ-9 (*Y*), and Distress Thermometer (*M*) scores were included in the model, both indirect effects through increasing Distress Thermometer scores (*B*: 0.098, CI: 0.038–0.029) and direct effects of SMDS scores (*B*: 0.21, CI: 0.086–0.33, *p* = 0.011) on PHQ-9 levels were significant. Higher SMDS scores led to higher PHQ-9 scores. When fear of COVID-19 (*X*), PHQ-9 (*Y*), and Distress Thermometer (*M*) scores were included in the model, the indirect effect through increasing Distress Thermometer scores (*B*: 0.133, CI: 0.045 to 0.23) was significant, whereas the direct effect of fear of COVID-19 scores on PHQ-9 levels (*B*: 0.083, CI: −0.06 to 0.23, *p* = 0.25) was non-significant.

When SMDS (*X*), GAD-7 (*Y*), and Distress Thermometer (*M*) scores were included in the model, the indirect effect through increasing Distress Thermometer scores (*B*: 0.112, CI: 0.034 to 0.20) was significant, whereas the direct effect of SMDS scores on GAD-7 levels (*B*: 0.057, CI: −0.053 to 0.16, *p* = 0.30) was non-significant. When fear of COVID-19 (*X*), GAD-7 (*Y*), and Distress Thermometer (*M*) scores were included in the model, both indirect effects through increasing Distress Thermometer scores (*B*: 0.14, CI: 0.044–0.23) and direct effects of Fear of COVID-19 scores (*B*: 0.21, CI: 0.016–0.26, *p* = 0.026) on GAD-7 levels were significant.

When SMDS (*X*), ASRS (*Y*), and Distress Thermometer (*M*) scores were included in the model, both indirect effects through increasing Distress Thermometer scores (*B*: 0.094, CI: 0.01–0.22) and direct effects of SMDS scores (*B*: 0.67, CI: 0.36–0.98, *p* < 0.0001) on PHQ-9 levels were significant. When Fear of COVID-19 (*X*), ASRS (*Y*), and Distress Thermometer (*M*) scores were included in the model, the indirect effect through increasing Distress Thermometer scores (*B*: 0.14, CI: 0.027 to 0.31) was significant, whereas the direct effect of fear of COVID-19 scores on ASRS levels (*B*: 0.33, CI: −0.03 to 0.68, *p* = 0.07) was non-significant. For all of the reported statistics, *B* scores represent the direction and magnitude of the effect.

## Discussion

This study investigated the change in depressive, anxiety, and attention deficit symptoms of college students who had previously applied to a university mental health center before the onset of COVID-19 in Turkey. Compared to the pre-pandemic period, students were found to have decreased symptoms of depression, anxiety, and attention deficit. Although the effect size was small for this change, the decrease in the rates of students with clinically moderate-to-severe depressive and anxiety symptoms was remarkable. Since high levels of stress, anxiety, and depression in college populations are a frequent finding in the literature, a modest decrease in symptomatology after the onset of the COVID-19 pandemic is a valuable finding. The decrease in stress levels and symptoms of social media use disorder were the predictors of this change, which is also another novel finding of our study. Although the screen time increased during the pandemic, the level of social media use disorder decreased. The levels of depression and attention deficit are affected by social media use disorder both directly and indirectly through stress level. The level of anxiety is affected by fear of COVID-19 both directly and indirectly through stress level. Therefore, the level of stress was mediating the relationship between (i) social media use disorder and depression, (ii) social media use disorder and attention deficit, and (iii) fear of COVID-19 and anxiety.

At the time of the recruitment for the study, the campus had been closed for approximately 3 months and the Turkish government had further implemented a number of social distancing measures. Despite many regulations restricting social life, the decrease in psychiatric symptomatology was an unexpected finding. The majority of studies reported increased mental health problems and a large-scale nationwide survey from China reported that massive media exposure and prior mental health problems were associated with an increased risk of college mental health problems in the early phase of the pandemic^15^. Only few studies such as a study from the USA conducted with Hispanic adolescents have reported decreased mental health problems, similar to our results^19^. Despite all its drastic effects, some individual stressors related to academic life such as rigid study timetables or social stressors such as the fear of missing out (FoMO) and competitive environment may be reduced at the time of crisis^35^ and these factors might be related to decreased stress levels in the early phase of the pandemic.

It is expected that the closure of the campus will result in disconnection from friends and romantic partners, thus creating risks of loneliness and isolation. However, in our sample, the majority of the students were living with their families or friends during lockdown which possibly minimized the negative effects of isolation. Even though the frequency of alcohol use and living with parents predicted the psychiatric symptoms in the first regression model, their effects became non-significant when social media use disorder and level of stress were added to the model. The reunion of the students, who stayed in the dormitories during the pre-pandemic period, with their families may have been beneficial in terms of social support and meeting basic needs. On the other hand, the fact that young adults may be asymptomatic carriers might have increased the students’ anxiety about the transmission of COVID-19 to their elderly family members^36^. Eventually, going back to the family house may increase social support and decrease household responsibilities but this protective effect might have been mixed with the stress of infecting the family members and possible adverse effects of the negative environment in some families. Of course, not every family is supportive and some families may have restrictive attitudes. There may also be concerns about covering online classes due to the lack of proper internet access and distraction due to a crowded family environment. A cross-sectional study from the USA reported that over 40% of the students were worried about their families who were more vulnerable and nearly half of them mentioned that their home was such a distractive environment^37^. Therefore, increased stress due to the pandemic can be compensated only to some extent by returning to the family home. The non-significant effect of living with family on mental health in this study might be a result of its mixed negative and positive effects.

According to mixed effect regression analysis, a decrease in stress and social media use disorder compared to the pre-pandemic period was found to be a significant predictor of a decrease in anxiety, depression, and attention deficit symptoms. This finding is in line with previous studies reporting that internet and smartphone addiction was associated with increased levels of depression during the pandemic^38,39^. Although several studies have shown a mediating effect of addictive social media use on anxiety^14,40^, social media disorder did not mediate the relationship between the decrease in stress levels and anxiety scores in this study. It can be predicted that the use of social media will increase in a period when physical interaction is restricted. In our sample, despite an increase in screen time, the decrease in social media use disorder rates can be explained by a possible change in the perception of social media use. Social media use behavior may have been somewhat normalized during the pandemic, and the possibility of perceiving intense use as pathological may have decreased. Moreover, increased screen time may be due to the time spent on online education, instead of the time spent on social media.

Compared to traditional media, social media has played a multitude of positive roles in information exchange during the COVID-19 crisis, including disseminating health-related recommendations, enabling connectivity and psychological first aid, showing public attitudes, experience, and perception of the disease as well as sentiment to the government^41,42^. Therefore, when more people use social media to seek and share health information, social media use can provide informational, emotional, and social support^43^. A Chinese study, which reported a similar rewarding effect of social media use, concluded that social media use helped to manage stressors and health risks. They suggested that this effect can be predicted by the informational, emotional, and peer support they received from the shared health information^44^. A recent study showed a dual impact of social media on mental health: using social media for reduced loneliness and entertainment was associated with poorer mental health, and using social media for personal contact and relationship maintenance was associated with better mental health^45^.

According to the mediator analysis, not the social media use disorder but the fear of COVID-19 mediated the relationship between stress and anxiety levels. Thus, low levels of COVID-19 fear yielded a decrease in anxiety both directly and indirectly by reducing the stress levels. This finding can be easily explained by a high correlation between fear of COVID-19 and general anxiety levels, which was already supported by the correlation analysis. Studies investigating the fear of COVID-19 often reported that it is a significant determinant of participants’ anxiety levels^46,47^.

Except for the problematic social media use, we could not show any effect of measured vulnerability factors on stress level. There are variables that were not assessed in this study and are still thought to be possible determinants of a reduction in stress levels. These may include the elimination of peer pressure and attendance obligation as well as moving away from the academic competition. Despite the high uncertainty, deadly infection concerns and limited social life at the beginning of the pandemic period; factors such as eliminating physical participation in classes, updating the grading system at Koç University as pass-fail under pandemic conditions, and eliminating the negative consequences of peer interaction may have caused students to perceive stress less compared the active school period. Actions that are taken by professors, such as reducing course loads, open book exams, and other allowances on grading requirements, may also have contributed to alleviate or reduce stress. For students receiving counseling services on campus, no access to those services could lead to exacerbation of psychiatric symptoms. Therefore, prompt telecommunication with students and warranting the continuation of mental health services such as telemental health services can prevent an increase in anxiety.

This study has several limitations. First, the observational research design does not allow us to make causal claims. Students tend to apply when their symptoms are most intense so the decrease in psychopathology may be related not only to the changing conditions during the pandemic but also to certain interventions before the pandemic. In fact, some symptoms might decrease over time. Although symptoms are likely to subside in some way prior to the pandemic, it has been hypothesized that a significant mental health-threatening stressor, such as a global pandemic, may be a triggering factor in a population with a prior history of psychiatric problems. Since the response rate was 50% and varied by gender and psychopathology severity, the attrition bias cannot be excluded. We ran an additional analysis to compare the baseline characteristics of respondents and non-respondents and did not find significant differences in terms of pre-pandemic psychiatric symptom scales between them. Second, the scales, used to measure dependent variables, are not diagnostic and are used for screening purposes. Third, relatively small sample sizes, sex imbalance, and the timing of the study (the study was conducted 3 months after the lockdown) limit the generalizability of our results. Because the pandemic is still unfolding, social isolation and economic impacts may have worsened over the past 1year. Moreover, the time period between the first and the last evaluation was not constant among participants. All the analyses were performed based on the mean score that subgroup analysis could not be performed due to the small sample size. Finally, not all stress factors such as social support and online education conditions were measured.

This study has several strengths such as longitudinal design and the development of regression models to measure the effect of risk factors. In addition, there were no differences among responders and non-responders in terms of psychiatric measurements, and the study was carried out in a well-planned timeframe according to the academic calendar, out of the exam period but before the final grades were submitted that academic concerns were still ongoing. There could be a relieving effect of termination of final exams; however, since the grades have not been announced yet, we cannot say the academic stress is over during recruitment.

Consequently, despite fear, uncertainty, and restrictions in the early stages of the pandemic, it may be possible to reduce stress and protect mental health with some interventions. These actions include promptly providing mental health services online and making adaptive arrangements for online education and grading systems, such as switching to the pass-fail system and encouraging the use of social media for communication and cooperation.

## Supplementary information

Supplementary information

Reporting Summary

Supplementary Table

## Data Availability

Data are available from the corresponding author on reasonable request.
