# Supplementary material for: Decreased psychiatric symptomatology after the onset of COVID-19 in a longitudinal college mental health study
Source: Npj Ment Health Res. 2022 Oct 21;1:17. doi: 10.1038/s44184-022-00017-4 (PMC9589876; doi:10.1038/s44184-022-00017-4)
Supplement: Supplementary file 3 — Supplementary Table [file 44184_2022_17_MOESM3_ESM.pdf]

Supplementary Table 1. Pre and Pandemic Health Behavior Assessments

	Pre-Assessment	Pandemic Assessment	p
**Body weight (mean±SD)**	64.96 ± 13.45	65.18 ± 14.05	0.56*
**Live with parents (yes/no)**	8/159	63/103	p<0.001**
**Problem falling asleep (yes/no)**	108/58	98/65	0.2**
**Sleep interruption (yes/no)**	73/93	53/110	p<0.001**
**Difficulty waking up (yes/no)**	135/31	116/47	p<0.001**
**Daytime sleep (yes/no)**	77/89	60/103	0.003**
**Sleepiness (yes/no)**	123/43	111/52	0.19**
**Screen time**			
**<1 hour**	8	3	p<0.001**
**1-3 hours**	61	16	
**4-6 hours**	75	81	
**>7 hours**	22	63	
**Number of days for alcohol use in last month**			
**0**	40	73	p<0.001**
**1-2**	42	35	
**3-5**	35	23	
**6-9**	30	14	
**10-19**	13	14	
**20-29**	6	4	

* : Wilcoxon Signed rank test

** : McNemar Test

Supplementary Table 2. COVID-19 Related Measurements (n=159)

**Fear of COVID-19 (mean±SD)**	17.23±5.43
**Experience of COVID-19 related symptoms**	
Yes	30
No	129
**Covid related symptoms**	
Did not experience any signs of infections	129
Had mild symptoms but did not admit to hospital	26
Admitted to hospital with mild symptoms and covid was excluded	3
Had a diagnosis of covid and recovered with outpatient treatment	1
**Number of going out/week**	
<3	73
3-5	63
>6	23
**Getting news from TV**	
Never	22
Some days briefly	72
Everyday briefly	38
Some days for a long period	23
Almost everyday for a long period	4
Getting news from internet	
Never	3
Some days briefly	42
Everyday briefly	85
Some days for a long period	26
Almost everyday for a long period	3
**Self-isolation Exposure other than housemates**	

Yes	71
No	88
**Company during the lockdown**	
Alone	6
With family	133
With friends	20

Supplementary Table 3. Frequency of pre-pandemic PHQ-9 scores

		Frequency	Percent	Valid Percent	Cumulative Percent
**Valid**	.00	7	4.2	4.2	4.2
	1.00	35	20.8	21.1	25.3
	2.00	50	29.8	30.1	55.4
	3.00	48	28.6	28.9	84.3
	4.00	26	15.5	15.7	100.0
	Total	166	98.8	100.0	
**Missing System**		2	1.2		
**Total**		168	100.0		

Supplementary Table 4. Frequency of follow-up PHQ-9 scores

		Frequency	Percent	Valid Percent	Cumulative Percent
**Valid**	.00	9	5.4	6.0	6.0
	1.00	63	37.5	41.7	47.7
	2.00	37	22.0	24.5	72.2
	3.00	29	17.3	19.2	91.4
	4.00	13	7.7	8.6	100.0
	Total	151	89.9	100.0	
**Missing System**		17	10.1		
**Total**		168	100.0		

Supplementary Table 5. Frequency of pre-pandemic GAD-7 scores

		Frequency	Percent	Valid Percent	Cumulative Percent
**Valid**	.00	15	8.9	9.0	9.0
	1.00	52	31.0	31.3	40.4
	2.00	48	28.6	28.9	69.3
	3.00	51	30.4	30.7	100.0
	Total	166	98.8	100.0	
**Missing System**		2	1.2		
**Total**		168	100.0		

Supplementary Table 6. Frequency of follow-up GAD-7 scores

		Frequency	Percent	Valid Percent	Cumulative Percent
**Valid**	.00	32	19.0	21.2	21.2
	1.00	64	38.1	42.4	63.6
	2.00	34	20.2	22.5	86.1
	3.00	21	12.5	13.9	100.0
	Total	151	89.9	100.0	
**Missing System**		17	10.1		
**Total**		168	100.0		
